# The Prognostic Value of Domain-Specific Cognitive Abilities Assessed by Chinese Version of Oxford Cognitive Screen on Determining ADLs Recovery in Patients with Post-Stroke Cognitive Impairment

**DOI:** 10.1155/2022/1084901

**Published:** 2022-09-06

**Authors:** Miaoran Lin, Jinxin Ren, Jingsong Wu, Jia Huang, Jing Tao, Lidian Chen, Zhizhen Liu

**Affiliations:** ^1^National-Local Joint Engineering Research Center of Rehabilitation Medicine Technology, Fujian University of Traditional Chinese Medicine, Fuzhou, Fujian, China; ^2^College of Rehabilitation Medicine, Fujian University of Traditional Chinese Medicine, Fuzhou, Fujian, China; ^3^Fujian Key Laboratory of Rehabilitation Technology, Fuzhou, Fujian, China; ^4^Key Laboratory of Orthopedics & Traumatology of Traditional Chinese Medicine and Rehabilitation (Fujian University of Traditional Chinese Medicine), Ministry of Education, Fuzhou, Fujian, China

## Abstract

**Background:**

Poststroke cognitive impairment (PSCI) has been increasingly recognized in patients. However, it remains unclear whether ADLs recovery is more susceptible to domain-specific cognitive abilities after a stroke. Therefore, the study was designed to investigate the cognitive functions of patients with PSCI at admission by using the Chinese (Putonghua) Version of the Oxford Cognitive Screen (OCS-P) as well as to identify the prognostic value of domain-specific cognitive abilities on the recovery of ADLs when discharged.

**Methods:**

A total of 153 hospitalized stroke patients were included in this prospective study. Cognitive function was assessed by OCS-P when participants were admitted to the hospital. The ADLs were measured at admission and discharge, and recovery was estimated by the improvement between admission and discharge. A diagnostic model using logistic regression was constructed to identify the prognostic value of domain-specific cognitive abilities for ADLs. The efficacy and accuracy of the diagnostic model were assessed by receiver operating characteristic (ROC) and Hosmer-Lemeshow's goodness of fit test. The diagnostic model was validated by 10-fold cross-validation and presented as a nomogram.

**Results:**

The score of OCS-P was 60(49.75, 69). The most frequently impaired cognitive domain was number writing (60.8%), followed by verbal memory (52.9%). Multivariate logistic regression showed executive dysfunction was a risk prognostic factor of ADLs recovery (*P* < 0.001, OR = 3.176 [95% CI, 1.218∼8.278]). The ROC curve of the diagnostic model was 0.839, with a good diagnostic efficacy. Hosmer–Lemeshow test showed diagnostic model had good calibration ability (*χ*^2^ = 8.939.3, *P*=0.347 > 0.05). The average error rate after adjustment of 10-fold cross-validation was 20.93%, within the acceptable range.

**Conclusions:**

Post-stroke patients generally suffered from multidimensional cognitive impairments. Executive dysfunction screened with OCS-P at clinical admission was a reliable and accessible predictive factor ADLs recovery in patients with PSCI. Early targeted rehabilitation programs are suggested to make them as earlier as possible, especially for those having executive dysfunction while hospitalized.

## 1. Introduction

Stroke is increasingly recognized as a major leading cause of disability, with high morbidity and mortality rates worldwide [1]. It's also known as a risk factor for cognitive impairment [2]. About 50% of stroke survivors have post-stroke cognitive impairment (PSCI) in the early stage [3], even in those with successful clinical recovery [4]. Up to 10% may develop dementia soon after the first stroke [5]. It directly interferes with patient's ability to perceive and adapt to the external environment, leading to adverse functional outcomes including but not limited to poor ADLs [6].

It was well established that PSCI can negatively affect ADLs, which now being considered a critical rehabilitation outcome to ensure the quality of life after stroke [7, 8]. A previous study had demonstrated that the global cognitive ability screened by Montreal Cognitive Assessment Scale (MOCA) in the early stage was positively associated with ADLs when they were discharged [9] and can predict long-term neurological recovery, ADLs recovery, even the mortality after stroke [10]. Similarly, Minimal Mental State Examination (MMSE) scores on admission were also identified as a predictor of functional outcomes after stroke [11]. PSCI generally involves impairments in different cognitive domains, concerning cognitive neglect [12], apraxia [13], aphasia [14], abstract reasoning [15], and executive dysfunction [16]. However, it remains unclear whether ADLs recovery is more susceptible to domain-specific cognitive abilities and the results had not been consistent.

Besides, it is important to accurately detect cognitive impairments in stroke rehabilitation. Therefore, a valid measurement specific for the identification of cognitive deficits in post-stroke survivors is critical for effective stroke rehabilitation treatments. Whereas, the most used existing instruments reported in previous studies were MoCA and MMSE [17, 18], which were not specifically targeted for stroke individuals and consequently had certain limitations in evaluating PSCI. Indeed, several studies have reported the flaws of these instruments in assessments of PSCI. Emerging evidence has indicated that MMSE cannot sensitively identify the impairment of abstract reasoning, executive ability, visual perception, and construction ability after stroke [19]. Although MoCA was thought to be more sensitive than MMSE [20–22], it still lacks specificity when patients have common cognitive conflicts after stroke, such as visual impairment, visual neglect, aphasia, or dyslexia [6, 22, 23]. For instance, stroke survivors with aphasia are unable to finish non-verbal tests (like Memory) of MOCA or MMSE. Similarly, those with unilateral spatial neglect usually fail to pass the alternating trail-making test, which may affect the authenticity of the test and its accurate evaluation of functional recovery [21].

Conversely, the Oxford Cognitive Screen (OCS), specially designed according to the characteristics of stroke, is superior to other cognitive screening tools, including MoCA and MMSE [24], for it allows a more precise assessment for common special cognitive impairment domains of stroke, such as aphasia, hemiplegia and spatial neglect and reduce any confounds that may occur because of these often-co-occurring difficulties, whereas the latter cannot. The Chinese (Putonghua) OCS (OCS-P) was the first Chinese version revised in our preliminary study [25]. It was translated in accordance with the requirements of the Chinese language. Moreover, language (semantics/picture pointing, sentence reading, and picture naming subdomains) and memory (free recall and recognition of verbal memory subdomains) domains were modified to accommodate the specificity of Chinese culture, while maintaining their equivalence to the content of the original version. The results revealed that OCS-P has satisfactory content validity, substantive validity, construct validity, inter- and intra-rater reliability, and known group discrimination. It was recommended on the official website of the Oxford Cognitive Screening Scale as a standardized clinical instrument specifically designed for measuring cognitive deficits of Chinese post-stroke patients [26]. In this study, OCS-P was used to comprehensively assess the cognitive function of patients with PSCI.

PSCI has been increasingly recognized in patients and has been proved to be closely related to the recovery of ADLs. However, it's not well explored whether ADLs recovery is more susceptible to domain-specific cognitive abilities after stroke when screening with OCS-P. Furthermore, an early diagnosis of cognitive dysfunction after stroke may have a great significance for the formulation of effective rehabilitation programs on ADLs recovery. Therefore, the study was conducted to identify the prognostic value of domain-specific cognitive abilities assessed by OCS-P in determining the ADLs recovery in patients with PSCI.

## 2. Materials and Methods

### 2.1. Study Design and Participants

This was a longitudinal prospective and explorative study. Patients from two tertiary hospitals were consecutively enrolled in the study according to the following inclusion criteria: (1) aged ≥18 years; (2) diagnosed as stroke and confirmed by CT or MRI [27]; (3) within 2 months after the first stroke; (4) MoCA scores <26; (5) volunteer to participate and sign the informed consent. Those with one of the following situations were excluded: (1) having another stroke during a hospital stay; (2) with a history of traumatic brain injury or degenerative brain disease; (3) diagnosed with dementia; (4) being unconscious or having unstable vital signs; (5) unable to complete the evaluation due to severe aphasia or dysarthria. All participants received routine standard rehabilitation treatment during hospitalization and those who could not adhere to rehabilitation treatment were excluded.

### 2.2. OCS-P Measurement

Cognitive function was assessed by OCS-P. OCS is a first-line, stroke-specific, and domain-specific cognitive screening tool for the identification of PSCI. It was developed by Demeyere at the University of Oxford, following rigorous psychometric and neuropsychological approaches [24]. It is composed of five domains (language, praxis, number, memory, spatial, and controlled attention) and these domains are further subcategorized into ten subscales. The core aim of making these tools available was to improve cognitive screening practices to detect cognitive changes, with a particular focus on vascular cognitive impairments. The modified version of OCS-P in our preliminary work was shown to have good reliability and validity [25].

### 2.3. ADLs Assessment

The primary outcome was the ADLs at discharge measured by Modified Barthel Index (MBI), ranging from 0 to 100 points, with a higher score corresponding to a greater ability to complete the ADLs. Participants were categorized into 2 mutually exclusive groups according to MBI scores: good outcome (0∼60) or poor outcome (60∼100) when they were discharged. The recovery of ADLs was identified by the improvement between admission and discharge.

### 2.4. Motor Function Assessment

Motor function was assessed by the Fugal–Meyer Assessment scale (FMA). FAM is an instrument commonly administered by physical therapists in both clinical and research fields to evaluate people after stroke. The original scale, which consisted of five domains (motor function, balance, sensation, joint mobility, and pain), has undergone rigorous investigations for reliability, validity, and responsiveness to change [28].

### 2.5. Neurological Recovery Assessment

The recovery of Neurological function was evaluated by the modified Rankin Scale (mRS). mRS is the most prevalent functional outcome measure in contemporary stroke research [29], with the scores ranging from 0 (asymptomatic), 1 (having a symptom but without obvious disability), 2 (mild disability), 3 (moderate disability), 4 (moderate to severe disability), and 5 (severe disability) to 6 (death).

### 2.6. Depression and Rehabilitation Participation

In addition, Hamilton Depression Rating Scale (HAMD) and Pittsburgh Rehabilitation Participation Scale (PRPS) were used to evaluate depression and rehabilitation participation respectively.

### 2.7. Data Collection and Procedure

The demographic information, clinical data, and functional status of participants were collected after hospital admission. Among these measurements, ADLs were assessed both after hospital admission and at discharge. Informed consent was obtained from all individual participants included in the study.

### 2.8. Statistical Analysis

The data was analyzed by SPSS 22.0 and R software. Two-sided *P* values of 0.05 were considered statistically significant. Measurement data were described by mean (standard deviation) or median and quartile rang [M (P25, P75)] for normal distribution or skewness distribution respectively. Enumeration data were presented as frequencies and percentages. We compared the recovery of ADLs among patients with different baseline demographic characteristics by using independent samples *t* tests or Mann-Whitney test for 2-level variables and the one-way ANOVA or Kruskal–Wallis test for variables with 3 or more levels. Pearson's or Spearman rank correlation analyses were used to demonstrating the correlations between clinical variables, cognitive function, and ADLs recovery. Multivariate logistic regression analysis was conducted to determine the prognostic value of domain-specific cognitive abilities for ADLs, with MBI score at discharge as the dichotomous outcome (0 = 60∼100, 1 = 0∼60) while significant variables from univariate and correlation analysis above as independent variables. The receiver operating characteristic curve (ROC curves) was used to measure the diagnostic effectiveness of the prediction models. The Hosmer Lemeshow goodness-of-fit test was used to evaluate the calibration capability of the prediction models. Prediction models were validated by *K*-fold cross-validation (*K* = 10). The nomogram was made to provide a more simple and convenient method for estimating the ADLs outcome.

## 3. Results

### 3.1. Sample characteristics

A total of 303 stroke patients were recruited from two tertiary hospitals and 148 of them were excluded for several reasons: did not meet the inclusion criteria (*n* = 86); eligible for exclusion criteria (*n* = 41); unwilling to participate (*n* = 21); dropped out because of weakness and fatigue (*n* = 2). Finally, 153 patients with PSCI were enrolled in our study and completed all the evaluations. Sample characteristics including demographic data, clinical data, and functional status were presented in Tables 1 and 2.

### 3.2. The Recovery of ADLs and Its Influencing Factors

The MBI scores of patients at admission and discharge were 30 (20, 47.5) and 60 (36.5, 80), respectively. The improvement of MBI was 20 (10, 35.5). Stroke subtype, hospital stays, ADLs at admission and neurological function were the influencing factors of ADLs recovery identified by using univariate and correlation analysis in Tables 1 and 2.

### 3.3. Cognitive Function of Patients with PSCI and Its Correlation with ADLs

The score of OCS-P was 60 (49.75, 69), with 94.12% of participants having at least one cognitive domain impairment. The highest frequency of the number of impaired cognitive domains was 4 (19.61%), while the lowest was 9 (1.31%). The most impaired cognitive domain was number writing (60.8%), followed by verbal memory (52.9%), the least impaired domain was the visual field (13.7%). The scores of cognitive domains of OCS-P and its correlations with ADLs were shown in Table 3. As a domain-specific cognitive function screened by OCS-P, executive function was shown to be a significant correlation with ADLs recovery.

### 3.4. Prognostic Value of Domain-Specific Cognitive Abilities for ADLs

**Figure 1:**
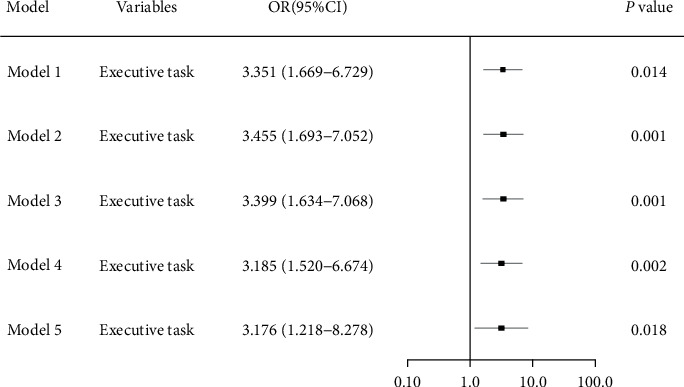
Forest plots of the prognostic value of executive function on ADLs (Model 1∼5). Model 1 was unadjusted; Model 2 was adjusted for demographic data (education years); On the basis of model 2, model 3 was adjusted for disease characteristics (type of stroke, hospital stays); Model 4 was mainly considered about the level of participation and adjusted for PRPS based on model 3; Model 5 considered the functional level at admission, so the MBI, MRS, and FMA at admission were adjusted based on Model 4.

**Figure 2:**
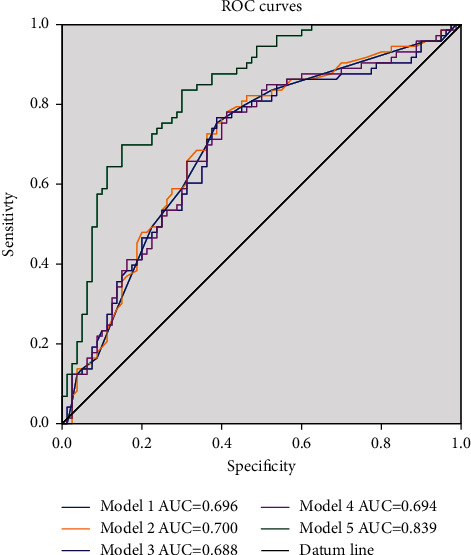
The area under ROC curves of five models. AUC: area under curves. The 95% CI of 5 models were model 1 (0.611∼0.780), model 2 (0.616∼0.784), model 3 (0.603∼0.773), model 4 (0.609∼0.778), model 5 (0.777∼0.902), with all *P* value <0.001.

**Figure 3:**
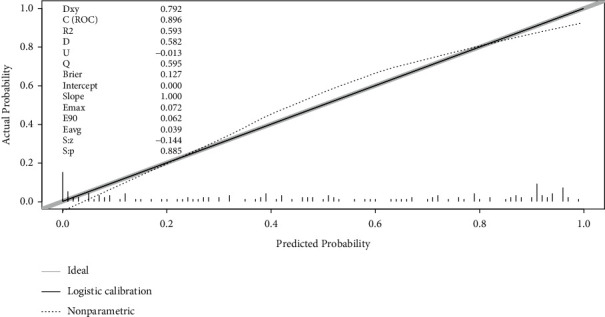
The calibration curve of model 5. The black line is the calibration curve. The gray line is the standard curve (*y* = *x*), indicating that the predicted number is the same as the actual observation number. The closer the two curves are, the better the calibration capability of the model.

**Figure 4:**
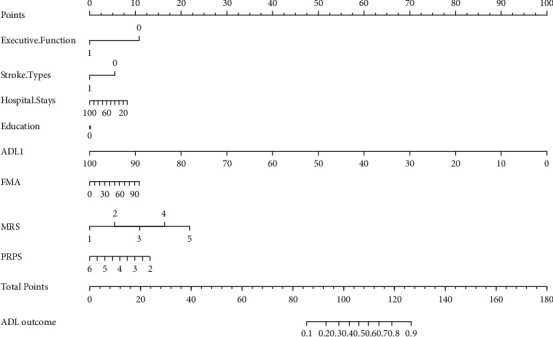
Nomogram of Model 5 to predict the ADLs for patient s with PSCI. ADLs1, the activity of daily living at the admission. FMA, Fugal–Meyer assessment scale. MRS, modified RNAKIN scale. PRPS, the Pittsburgh rehabilitation participation scale. Executive function: 1 = normal, 0 = abnormal. Stroke types: 0 = hemorrhage, 1 = ischemia. ADLs outcome: 0 = good outcome (0∼60), 1 = poor outcome (60∼100). Each clinical relative factors corresponds to a specific point by drawing a line straight upward to the points axis. After the sum of the points is located on the total points axis, the sum represents the probability of ADLs by drawing straight down to the ADLs outcome axis.

The significant variables in Tables 1 and 2 and being reported as important factors (education years, PRPS, FMA) which can affect ADLs [30] were introduced into the multivariate logistics regression equation in sequence to identify the prognostic value of domain-specific cognitive ability (executive function in Table 3) on determining the ADLs recovery in patients with PSCI, generating five models in total. The forest plots of five models were presented in Figure 1. The area under curves of ROC curves of five models was presented in Figure 2, with the Model 5 having the best diagnostic performance (area under curves = 0.839). The multivariate logistic regression analysis of Model 5 showed executive dysfunction was a risk prognostic factor of ADLs recovery (*P* < 0.001, OR = 3.176 ([95% CI, 1.218∼8.278]) in Table 4. No statistical significance was found between predictive value and actual observed value of Model 5 (Hosmer-Lemeshow *χ*^2^ = 8.939.3, *P*=0.347 > 0.05), indicating the model has good calibration ability. The calibration plot and nomogram of Model 5 were shown in Figures 3 and 4. Besides, the model 5 was validated by *K*-fold cross-validation. The data was randomly and equally divided into 10 groups, 9 of which were used for modeling, and the others were used for verification. After 10 times of modeling and verification in sequence, a relatively stable model was achieved. The average error rate after the correction was 20.93%, within the acceptable range.

## 4. Discussion

Cognitive impairment is a so common dysfunction existing in post-stroke patients which cannot be overlooked. In this study, we primarily used OCS-P, a more targeted measurement we specially revised for stroke patients in our preliminary study, to identify the prognostic value of domain-specific cognitive abilities on the recovery of ADLs in patients with PSCI. The result showed that post-stroke patients generally suffered from multidimensional cognitive impairments and executive function screened with OCS-P at clinical admission was a reliable and accessible predictive factor of ADLs. Our findings emphasized it's important for medical staff and rehabilitation therapists to focus on the executive dysfunction of stroke inpatients as early as possible.

The original OCS has been designed as a cognitive screening tool that acts as a pointer for further, more detailed domain-specific assessment should impairments in any cognitive domain be revealed. The common clinical instruments used by clinicians in neurology, rehabilitation medicine, and therapy mostly quantify general cognitive function of patients. Test constructs of these instruments such as MMSE and MOCA cannot capture the cognitive challenges unique to post-stroke patients. Unlike these current screening tools, OCS allows assessment of dysplasia patients, even can be delivered at the bedside in acute stroke. Moreover, OCS provides measures of neglect (both allocentric and egocentric), praxis, and numerical cognition. The test items were presented both visually and verbally, inclusive for the possibility of selecting a correct answer from a multiple-choice array.

Currently, OCS has been translated into several versions, including Hong Kong (Cantonese speaking) [31], Italian [32], and Russian versions [33]. The modified Chinese (Putonghua) version of OCS-P was considered to have good psychometric properties in our previous research. It was validated for use by clinicians in China and among other cultural groups (or individuals) living outside of China. Clinicians and researchers can administer OCS-P to patients who speak Chinese (Putonghua) at admission and during subsequent follow-up assessments [26]. The OCS-P subscale scores can be used for guiding treatment plan, monitoring treatment effect, and tracking rehabilitation outcomes. In this current study, it had been proved to have a good prognostic value for ADLs of stroke survivors with PSCI.

Early findings including our own have demonstrated that cognitive impairment was highly prevalent in patients after stroke. According to our study, 94.12% of the patients had at least one cognitive impairment, and 85.62% of them had at least two. It was basically confirmed in previous studies, which demonstrated that nearly 86% [21] and 91.6% of the post-stroke patients have at least one impaired task in the cognitive field after stroke by using the OCS scale and more than 80% of them had two or more [34]. The percentage of impaired cognitive domains exceeding 50% among ten cognitive domains of OCS-P were: number writing (60.8%), verbal memory (free recall) (52.9%), and executive function [executive task (mixed)] (50.3%). Demeyere used the OCS scale to assess patients with acute stroke and revealed that executive dysfunction (48.9%), neglect (39.8%), and number writing (31.1%) were mainly impaired cognitive domains [21]. In Mauro's study, cognitive impairment mainly occurred in calculation (50.7%), sentence reading (49.8%), number writing (36%), executive function (32.3%) and neglect (31.3%) [34]. Patients after stroke generally have multiple cognitive domains impairment. Although the impaired cognitive domains were inconsistent in early findings, most of them were mainly concentrated in executive dysfunction, neglect, and number writing, illustrating those domains were highly susceptible to stroke-related impairments.

PSCI has a negative impact on early activity limitations and participation restrictions [35]. Our study verified patients with executive dysfunction after the stroke had a 3.176 times risk of poor ADLs than those with normal executive function. Thus, the executive function of stroke survivors at admission should be considered a powerful independent predictor of ADLs when discharged. Similar results were demonstrated in recent works [9, 16, 36, 37], which collectively identified executive dysfunction as a significant and independent predictor of functional outcome. According to previous studies, executive function subtests of the OCS were reported to predict the long-term functional capabilities of post-stroke patients [37], higher initial executive function scores of MoCA were associated with better ADLs in the subacute stroke phase [9], and trail making test-A scores, as a measurement of executive function, can also highly predict the MBI score at discharge [16]. Even in follow-up, the inhibition of executive function was strongly associated with earlier permanent institutionalization and its prognostic value was also recommended after stroke [36]. Moreover, a prospective study with 7717 individuals revealed that compared to those without executive dysfunction, those with poor baseline executive function had significantly worse ADLs and instrumental activities of daily livings (IADLs) function cross-sectionally over 6 years and had an increased risk of mortality [38]. Another meta-analysis suggested a consistent moderate association between ADLs and executive function [39], supporting the growing evidence for a link between ADLs and executive dysfunction in early cognitive decline.

Executive function refers to a multidimensional goal-directed system, monitoring one's behavior and self-regulating functions [40, 41]. These processes empower us to effectively prioritize goals, weigh benefits and respond adaptively. As cognitive deficits progress, executive dysfunction becomes more prominent and its negative effect on instrument ADLs had also been shown to be an important contributor to the cognitive deterioration [42]. In our study, executive function as an important prognostic factor of ADLs may due to the following reasons: First, executive dysfunction did affect the individual's ability to effectively participate in rehabilitation programs, manifested by the inability to maintain a series of behavioral consistency, initiate actions, suppress impulsive behaviors, and follow the rehabilitation instructions [15]. Second, for stroke patients, executive dysfunction has been found to be related to an increased tendency to adopt avoidant coping strategies [43], which were positively associated with adverse outcomes. Third, executive dysfunction was associated with a lower level of participation [44]. Patients with more worse executive function appear to have a low quality of participation, leading to poorer functional recovery and increased hospitalization time. These results collectively suggest that screening of executive dysfunction by OCS-P can help to identify those at risk for loss of ADLs ability.

Except for executive function, the study did not find other cognitive domains of OCS-P that can be served as a predictor of ADLs, which was inconsistent with some previous studies [9, 45]. This may be due to the study design factors, such as sample size, participant's age, or follow-up period [35]. It needs to be further confirmed in longitudinal studies based on a large population. Besides, our results did not support the previous findings that overall cognitive function has a good predictive value for the recovery of ADLs [10,46]. In our study, we used the number of task impairments of the OCS-P scale as overall cognitive function [24] and no correlation was found with ADLs outcome. However, this finding needs to be further explored with a larger sample size.

The study has several limitations. First, most patients in our study were transferred from general hospital to rehabilitation hospital. They were generally thought to be more seriously ill and this selection bias may lead to an overestimation of the incidence of cognitive impairment after stroke. Moreover, due to the small sample size and single-center study design, which included only Chinese hospitalized post-stroke patients, it should be cautious when we generalized our findings. Finally, the observation time was only limited to the rehabilitation period of stroke patients from admission to discharge. There may be biases in assessing the frequency of task impairments. Future research with larger sample sizes from multiple centers and longer observation time exploring the diagnostic value of cognitive impairment on short-term and long-term ADLs recovery of stroke patients are expected.

## 5. Conclusion

Executive dysfunction screened with OCS-P at clinical admission was a reliable and accessible predictive factor of ADLs recovery in patients with PSCI. Early targeted rehabilitation programs are recommended to take especially for those who have executive dysfunction while hospitalized.

## Figures and Tables

**Table 1:** Sample characteristics and the univariate analysis of ADLs recovery.

Variables	*N* (%)/*M* (P25, P75)	Improvement of MBI mean (SD)	*P* value
Gender^#^			0.888
Male	111 (72.5%)	24.54 (19.92)	
Female	42 (27.5%)	22.81 (15.72)	
Age (years)^a^^*∗*^	63 (53.5, 70)		0.325
≤63	47 (54, 59)	25.51 (20.01)	
>63	66 (70, 75)	22.44 (17.38)	
Smoking history^b^			0.362
Yes	38 (24.8%)	26.63 (19.23)	
No	115 (75.2%)	23.22 (18.69)	
Drinking history^#^			0.738
Yes	38 (24.8%)	24.16 (18.68)	
No	115 (75.2%)	23.79 (19.50)	
Stroke subtype^#^			0.026
Ischemia	103 (67.3%)	21.62 (17.39)	
Hemorrhage	50 (32.7%)	29.10 (20.77)	
Brain injured area^#^			0.713
Right	79 (51.6%)	23.46 (17.07)	
Left	52 (34.0%)	24.96 (18.97)	
Both	22 (14.4%)	24.14 (24.58)	
Atrial fibrillation^#^			0.756
Yes	13 (8.5%)	21.46 (16.57)	
No	140 (91.5%)	24.31 (19.05)	
Hypertension^#^			0.486
Yes	51 (33.3%)	22.61 (18.06)	
No	102 (66.7%)	24.79 (19.24)	

^a^Subjects were divided into younger age group and older age group according to the median age (63 years old).^b^Quit smoking and still smoking were considered to have a history of smoking while never smoking was considered as no smoking history. Like smoking history, quit drinking and still drinking were considered to have a history of drinking while never drinking was considered as no smoking history. ^#^Two independent sample *t* tests or analysis of variance (ANOVA). ^*∗*^Nonparametric rank sum Z test (Mann-Whitney test).

**Table 2:** Sample characteristics and the correlation analysis of ADLs recovery.

Variables	Mean (SD)/*M* (P25, P75)	*r*	*P* value
Education years	7 (4, 10)	0.099	0.222
BMI	22.49 (20.76, 24.8)	0.081	0.341
Course of disease	22 (11.75, 30)	0.112	0.173
Hospital stays	37 (26, 59.5)	0.197	0.015
Fasting plasma glucose	5.35 (4.65, 6.25)	0.064	0.429
Triglycerides	1.47 (1.06, 1.81)	−0.131	0.106
Total cholesterol	3.7 (0.99)	−0.008	0.920
FMA	18 (10, 43)	−0.075	0.356
HAMD	5 (3, 7)	−0.046	0.568
PRPS	4.75 (4, 5.25)	0.047	0.563
MRS	4 (4, 4)	0.242	0.003
MBI at admission	30 (20, 47.5)	−0.235	0.003

BMI, body mass index; MBI, modified barthel index; FMA, fugal-meyer assessment scale; HAMD, hamilton depression rating scale; PRPS, pittsburgh rehabilitation participation scale; MRS, modified rankin scale.

**Table 3:** The cognitive function of participants and its correlation with ADLs recovery.

Domains	Tasks	*M* (P25, P75)	Impaired percentage (%)	*r*	*P* value
Number of cognitive impairment tasks		4 (2, 6)		0.022	0.786

Attention	Executive task	3 (−1, 5)	35.3	−0.22	0.006
	Executive task (mixed)	6 (4, 11)	50.3	0.049	0.546
	Visual field test	4 (4, 4)	13.7	−0.004	0.961

Language	Semantics/picture pointing	3 (3, 3)	18.3	0.061	0.456
	Sentence reading	18 (11.75, 19)	28.7	−0.13	0.113
	Picture naming	3 (2, 4)	36.6	−0.058	0.479

Memory	Orientation	4 (3, 4)	19	0.013	0.876
	Verbal memory: free recall	2 (1, 4)	52.9	0.108	0.186
	Verbal memory: recognition	3 (2, 4)	35.9	0.022	0.789

Number	Number writing	2 (1, 3)	60.8	0.042	0.603
	Calculations	4 (3, 4)	16.3	−0.021	0.801

Spatial neglect	Broken hearts test (gap)	0 (0, 1)	41.4	−0.011	0.892
	Broken hearts test (complete)	0 (−1, 2)	45.1	0.029	0.724
Praxis	Meaningless gesture imitation	9 (7, 11)	29.4	0.121	0.137

**Table 4:** Multivariate logistic regression analysis of model 5.

Variables	*B*	S.E	Or (95% CI)	*P* value
Executive function	1.156	0.489	3.176 (1.218∼8.278)	0.018
Education	0.001	0.056	1.001 (0.897∼1.116)	0.990
Hospital stays	−0.010	0.010	0.990 (0.970∼1.011)	0.349
Stroke type	0.589	0.531	1.802 (0.637∼5.100)	0.267
PRPS	−0.353	0.285	0.703 (0.402∼1.227)	0.215
FMA	0.012	0.014	1.012 (0.984∼1.040)	0.411
MRS	0.585	0.668	1.794 (0.485∼6.642)	0.381
MBI at admission	−0.107	0.020	0.898 (0.864∼0.935)	<0.001

MBI, modified barthel index; FMA, fugal-meyer assessment scale; MRS, modified rankin scale; PRPS, pittsburgh rehabilitation participation scale.

## Data Availability

The data that support the findings of this study are available upon reasonable request to the corresponding author.
